# Supplementary material for: Early detection of complications in pancreas transplants by microdialysis catheters, an observational feasibility study
Source: PLoS One. 2021 Mar 11;16(3):e0247615. doi: 10.1371/journal.pone.0247615 (PMC7951931; doi:10.1371/journal.pone.0247615)
Supplement: S1 File — The protocol for the project, in which the microdialysis part is described in part 2.7. (DOC) [file pone.0247615.s001.doc]

Clinical Study Protocol

A prospective, observational study in Pancreatic Allograft Recipients:

The effect of risk factors, immunosuppressive level, and the benefits of scheduled biopsies and microdialysis monitoring – on surgical complications, rejections and graft survival.

Study number: OUS-PTx-01

### Final Protocol Date: 2-May-2013

Final Amendment Protocol date: 20-Nov-2014

We have read the protocol of this study and confirm that all information necessary to conduct the study is provided by these documents. We are prepared to perform the study according to this protocol

**Investigator**

______________________ _______________________ ___/___/___

Print Name Signature Date

**Co-Investigators**

______________________ _______________________ ___/___/___

Print Name Signature Date

______________________ _______________________ ___/___/___

Print Name Signature Date

______________________ _______________________ ___/___/___

Print Name Signature Date

TABLE OF CONTENTS

1 INTRODUCTION AND BACKGROUND 4

1.1 The Norwegian experience 4

1.2 Immunosuppressive therapy 5

1.3 Rejections/Donor-specific antibodies (DSA) 6

1.4 Graft monitoring with microdialysis catheters 6

2 OBJECTIVES 7

2.1 Primary objectives 7

2.2 Secondary objectives 7

2.3 Immunological studies 8

2.4 Endoscopic mucosal imaging and ultrasound 9

2.5 Donor and recipient baseline characteristics 9

2.6 Non-immunological rejection markers 9

2.7 Microdialysis monitoring 9

3 STUDY DESIGN 10

4 DURATION OF STUDY 11

###### 5 NUMBER OF PATIENTS 11

6 SELECTION OF PATIENTS 11

6.1 Inclusion Criteria 11

6.2 Exclusion Criteria 11

7 DOSAGE AND ADMINISTRATION 11

7.1 Immunosuppression 11

7.2 Concomitant Treatments 12

8 TREATMENT OF ACUTE REJECTION EPISODES 13

9 ADVERSE EVENTS 13

9.1 Definitions 13

9.2 Serious Adverse Events 14

9.3 Serious Unexpected Adverse Events 14

9.4 Follow Up of Adverse Events 15

10 ANALYSIS OF RESULTS 15

10.1 Strategy regarding 0-hypothesis 15

10.2 Sample size and Power analysis 16

10.3 Statistical methods in data analysis 17

10.4 Provisional analysis 17

### 11. DATA HANDLING AND RECORD KEEPING 18

11.1 Case Report Form 18

11.2 Record Retention 18

12. ETHICAL CONSIDERATIONS 18

12.1 Institutional Review Board (IRB)/Ethics Committee (EC) 18

12.2 Informed Consent 18

12.3 Declaration of Helsinki 18

12.4 Good Clinical Research Practice (GCP) and 18

12.5 Unanticipated Problems 18

13 PUBLICATIONS 18

14 REFERENCES 19

15 STUDY FLOW CHART 21

**1 INTRODUCTION AND BACKGROUND**

The first pancreas transplantation (PTx) was performed in Minnesota in 1966 by Kelly

and colleagues (1). In recent years the number of procedures has grown considerably worldwide, and is now a well-established treatment option for patients with diabetes mellitus with and without concomitant diabetic End-Stage Renal Disease (ESRD) (2-4). The indication for PTx is advanced and/or badly controlled diabetes mellitus (”brittle” diabetes, severe hypoglycemic episodes,”unawareness”, etc). Solitary pancreas transplantation (SPT; without concomitant kidney transplantation) is usually classified as PTx alone (PTA), PTx after kidney transplantation (PAK) or PTx after islet transplantation (PAI). Kidney transplantation of the diabetic uremic population increases survival compared to long-term dialysis (5, 6). Transplant options for patients with diabetic end-stage nephropathy include simultaneous pancreas-kidney (SPK), live donor kidney (LDK) and deceased donor kidney (DDK) transplantation. SPK transplantation relieves not only the patient’s uremia, but also alleviates the hyperglycemic state of diabetes. Large international patient registries show that patient survival rates after SPK have reached more than 95% at 1 year and 87% at 5 years post-transplant, respectively (2). Nevertheless, PTx as treatment for type 1 diabetes has not gained the same popularity as transplantation of other organs, partly because PTx have been associated with a high rate of surgical complications; particularly bleeding, thrombosis and exocrine leakage. Furthermore, there has been a lack of reliable, non-invasive rejection monitoring instruments, and the invasive, percutaneous pancreas biopsies have traditionally been associated with a high rate of complications (exocrine leakage, bleeding).

The difficulties encountered with PTx have to some extent been compensated by a very selective attitude towards the donors, but thereby making pancreas grafts a scarce resource. In contrast to other abdominal transplantations such as liver transplantation (LTx) and kidney transplantation (KTx), where repeated biopsies have been used for immunosurveillance, percutaneous biopsies of the pancreas-graft have traditionally been avoided due to a high rate of biopsy-related complications (exocrine leaks/fistulas and bleeding episodes). Thus, fear of acute rejections and lack of adequate rejection markers, have led to a rather intensive immunosuppressive load in PTx recipients.

Solitary pancreas transplantation (SPT) has traditionally been subjected to even higher complication and rejection rates, with inferior graft and patient survival - thus favoring the combined SPK procedure. This has been attributed to an even worse rejection monitoring capability, without a”reporter”/sentinel allograft kidney. No biochemical markers have proven to be effective in rejection surveillance.

Pancreas graft thrombosis is a feared complication in the postoperative course, partly due to the oversized vessels used (coeliac trunk/superior mesenteric artery/portal vein) in conjunction with the low blood flow through an isolated pancreas graft. In the native setting, these vessels also serve the intestines and spleen. Therefore, PTx poses a delicate balance between thrombosis and bleeding complications.

*End stage type 1 diabetes is a devastating chronic disease. PTx offer long term insulin-independency. Efforts should be made to define robust patient selection criteria and offer eligible patients insulin-independency before severe diabetic complications appear.*

**1.1 The Norwegian experience**

PTx is performed at one single national center in Oslo, and from 1983 to date 300 procedures have been performed (7-11). In recent years, the activity has increased; 22 were performed in 2011 and 28 in 2012. Approximately 9 out 10 PTx’s have been SPK’s, hence only about 10% have been SPTs. In the first period from 1983 through 1987, a duct-occluded segmental pancreas was used for transplantation. From 1988, the whole pancreas graft was used, and the exocrine secretion was drained by anastomosing the duodenal segment to the urinary bladder. This technical solution was chosen partly because it offered some sort of rejection monitoring, by urine amylase counts and cystoscopic pancreas biopsies. However, many patients suffered from chemical cystitis and metabolic acidosis, due to loss of bicarbonate. In 1998 the urinary bladder anastomosis was abandoned, in favor of the more physiological enteric anastomosis, the duodenal segment being connected to the proximal jejunum. However, this solution offered even less options to monitor upcoming rejections, as percutaneous biopsies was mostly avoided due to the previously mentioned hazards.

We have recently examined (12) all PTx’s performed at our hospital during 2006-2010 (n=61; 59 SPK, 2 PTA). Our overall surgical complication rate has decreased from earlier years, but we still suffer a substantial rate of reoperations (about 30% of patients), mainly caused by exocrine leakage, bleeding and vein thrombosis. When comparing the populations with or without reoperation, higher donor age had a significant negative impact. No significant effect of donor age on graft survival was observed. There was a tendency towards better results in female recipients, both regarding surgical complications and graft survival. The rejection rate (altogether about 30%) was significantly higher in the graft loss group, and reoperations were insignificantly associated with graft loss.

From late 2011 and up to date, several measures have been implemented to improve outcome and reduce the rate of surgical complications. In line with most Tx centers in Scandinavia, we have switched the prophylactic anticoagulation treatment from our traditional Macrodex® regime to a Fragmin® regime. Several surgical/technical changes have also been implemented during recent years; tentatively more atraumatic graft procurement, preserving the entire coeliac arterial axis including the gastroduodenal artery, obtaining a long portal vein to reduce the need for elongation, as well as extended in situ dissection by means of Ligasure^TM^. Due to the conventional lack of rejection monitoring parameters, we launched an investigatory surveillance program, with protocol biopsies of the duodenal segment via double balloon enteroscopy (13). The impact and value of this program has yet to be investigated. Previous reports have described separate rejection of the pancreas or kidney in the SPK setting, and the gold standard for proving rejection of the pancreas is undoubtedly a biopsy of the pancreas itself. This encouraged us to further develop techniques for better surveillance, such as endoscopic transduodenal ultrasound-guided biopsies of pancreas (EUSBP). Inferior outcome of PTA and lack of valid tools for immunosurveillance in the absence of a simultaneous kidney graft, have led some centers to evolve the duodeno-duodenostomy (DD) for drainage of the exocrine pancreas, making the EUSBP possible. There are many theoretical advantages with the DD, especially regarding rejection surveillance, and we have recently adopted this technique. The endoscopic access afforded by the DD also makes it possible to stent the pancreatic duct in case of exocrine leakage.

Though, in recent years we have experienced a very low incidence of complications with the conventional percutaneous ultrasound-guided pancreas biopsy. In our latest, retrospective study (Horneland et al., Am J Transpl; in press), focusing on the duodenoduodenostomy, there were no complications among 18 percutaneous pancreas biopsies performed.

**1.2 Immunosuppressive therapy**

Over time, the induction therapy and maintenance immunosuppressive protocols have changed. From 1983 to 2000, all recipients received triple immunosuppressive regimens with cyclosporine, azathioprine and prednisolone (CS). During the last part of the 1990’s azathioprine was substituted by Mycophenolate mofetil (MMF), and cyclosporine was substituted by tacrolimus. After 2000, the immunosuppression has been intensified by induction therapy both for PTx (Antithymocyte globulin (ATG)) and for kidney transplants alone (basiliximab). Thus in recent years, PTx recipients have received a quadruple immunnosuppressive regimen, that includes tacrolimus, MMF, CS and ATG. The dosage of ATG has been directed by T-cell counts.

**1.3 Rejections/Donor-specific antibodies (DSA)**

During recent years, with the quadruple immunnosuppressive regimen, our biopsy-verified rejection rate has been about 30% (12).

In this study we will follow the routine protocol for treatment of rejections; primarily more CS (5-8 doses of SoluMedrol), secondarily more ATG (2-5 doses; T-cell directed)

A recent study (14) assessed the role of post-Tx HLA antibody monitoring in the surveillance of PTx recipients, and the impact of DSA. Four hundred thirty-three PTx’s were performed at the Oxford Transplant Centre (317 SPK/116 Sol-PTx). It was demonstrated that 39.8% of patients developed de novo HLA antibodies, of which 36.9% were de novo *DSA*. The development of antibodies to donor HLA, but not to nondonor HLA, was significantly associated with poorer graft outcomes, with 1- and 3-year graft survival inferior in SPK recipients, and interestingly even more so in Sol-PTx recipients. In a multivariate analysis, development of de novo DSA emerged as a strong independent predictor of pancreas graft failure.

These findings have urged us to investigate de novo DSA development in the present study.

**1.4 Graft monitoring with microdialysis catheters**

As stated in the previous paragraphs the complication rates following pancreas transplantation are high. Except maybe for severe hemorrhage, all complications have in common that they are difficult to detect. Accordingly, there is an emerging need for better monitoring of pancreas transplants. We consider that further improvement of surgical techniques and immunosuppressive protocols rely upon better monitoring tools.

Microdialysis is a technique, which enables close to ‘real time’ monitoring of tissues and organs of interest. Depending on the membrane’s pore size, metabolic substances (lactate, pyruvate, glucose and glycerol) and/or mediators of inflammation (cytokines, chemokines and complement factors) are sampled in a feasible way (15). So far, the method’s ability to detect brain ischemia is the best validated (16). In the United States clinical application is so far restricted to neurointensive care units, as only the brain catheter (CMA 70, CMA Microdialysis AB, Stockholm, Sweden) is approved by the Food and Drug Administration for clinical use. However, there are more than 2000 clinical reports on microdialysis catheters, and in Europe the catheters are Conformité Européenne marked for a wider range of indications.

We have done extensive clinical observation trials using microdialysis catheters in liver transplanted patients and we have inserted more than 200 catheters in hepatic tissue without experiencing any major complications (17-21).Graft thrombosis has been detected almost in ‘real time’ as elevated intrahepatic lactate and lactate to pyruvate ratio. Rejection has been detected several days before the rise in conventional blood markers (bilirubin and transaminases) by elevated lactate and with unchanged lactate to pyruvate ratio. Acute cellular rejections were detected with more than 80 % sensitivity and specificity. Ischemic complications like hepatic artery thromboses have been detected with 100 % sensitivity and specificity. We also revealed potentially specific biomarkers for ischemia (complement factor 5a) and rejection (CXCL-10) (19) . We have now implemented microdialysis as routine standard of care in *pediatric* liver transplants.

We are also investigating the potential role of microdialysis in monitoring patients who have undergone Whipple’s operation for pancreatic or duodenal cancer. Preliminary results show that leakages in the pancreaticojejunostomy can be detected at a very early time point, by increased concentrations of glycerol in samples collected from catheters positioned close to the enteroanastomosis.

**2 OBJECTIVES OF THE STUDY**

Several studies have shown acceptable results after PTx by substituting ATG with basiliximab (22-26), which is considered to convey a considerably lower number of adverse events. However, our experiences with ATG in PTx (introduced in 2004) are good, and our presumably gentle way of administrating the drug – directed by T-cell counts - is in fact unique (12). The potential advantages of reducing the overall corticosteroid (CS) load is obvious, as CS is a well-known pro-diabetic agent and causes severe long term adverse effects (22).

On this background, we have very recently reduced our CS dosing (in the routine protocol) to a level corresponding to our Kidney Tx protocol (valid since 2009). Thus*, we intend to prospectively investigate and compare a single cohort of our present PTx immunosuppressive protocol with previous (historical) cohorts.*

The rationale for the study is that; *i*) a high immunosuppressive load, and in particular CS, may be partly responsible for the high rate of PTx associated complications/reoperations; *ii*) a high immunosuppressive load is related to infectious complications; *iii*) improved PTx rejection surveillance by DD and EUSBP allows a lower-graded immunosuppressive protocol; iv) evaluating the surgical and medical measures made to our PTx program during the past two years

**2.1** **Primary objectives**

- Compare the incidence of *acute rejection episodes* at 6, 12, 36 and 60 months after pancreas transplantation, between our single prospective cohort with lower CS vs a historic, retrospective control group (PTx performed during 2011-2013). The incidence of rejection is defined as the fraction of patients in which rejections episodes (one or more) have been proven by biopsies. For SPK rejection in eitherorgan, pancreas or kidney, counts.
- Compare the incidence of *surgical complications*, involving reoperations and reinterventions, between the prospective study group and retrospective control group.

**2.2** **Secondary objectives**

- Compare the number and severity of rejection episodes in the pancreas allograft to the ones occurring in the kidney allograft (SPK), and the ones diagnosed by the duodenal segment biopsies.
- Compare pancreas graft survival at 12, 36 and 60 months after transplantation between the prospective study group and retrospective control group.
- Monitor kidney (and pancreas) graft survival (SPK) 12, 36 and 60 months post-Tx.
- Compare patient survival at 12, 36 and 60 months post-Tx.
- Compare the incidence of non-surgical complications (infections,cardial complications, pulmonary complications and neurological complications).
- Explore if potentially graft devastating complications in pancreas transplants can be detected earlier with the microdialysis method than by current standard of care
- Explore if new complication specific biomarkers can be detected by the microdialysis method

**2.3 Immunological studies**

- Scheduled endoscopic (EUS) biopsies will be taken according to our present routine protocol; simultaneously from these four transplant/organ sources at predestined points of time:

- Pancreas transplant (*P*)

- Kidney transplant (*K*)

- Duodenal segment of pancreas transplant (*tD*)

- Duodenum of recipient = native Duodenum (*nD*) – Serves as ‘control’

• Baseline (Day 0; at Tx): *K +tD + nD* - These will be taken during surgery

• 3 weeks post-Tx: *P +tD + nD* - Endoscopic

• 6 weeks post-Tx: *P + K + tD + nD* - Endoscopic (P/tD/nD) + Percutaneous (K)

• 12 months post-Tx: *P + K + tD + nD* - Endoscopic (P/tD/nD) + Percutaneous (K)

Within the study, we also want to take conventional percutaneous, ultrasound-guided P biopsies at 6 weeks and 12 months post-Tx – in order to compare the yield (and complications) by EUS vs percutaneous P biopsies.

In addition, indication biopsies will be taken whenever there is suspicion of rejection

in either organ. Preferably, simultaneous P + tD + nD endoscopic biopsies, P

percutaneous biopsies and K percutaneous biopsies should be obtained.

All biopsies will be examined at our local pathology unit; the pancreas and duodenal biopsies by prof. Tor Jacob Eide and dr. Krzysztof Grzyb; the kidney biopsies by prof. Helge Scott and dr. Erik Heyerdahl Strøm. The pancreas and kidney biopsies will be evaluated by well-known BANFF criteria, while duodenal biopsies will be rated according to Wu et al. (27).

- Histological evaluation of the scheduled and indication biopsies will involve comparisons between biopsies from the various transplants/organs of the same patient.
  - Rejection histology scores.
  - Immunoshistochemistry on immunologic markers (CD25, FOXP3, CD4, CD3, CD8, CD45RO, perforin, granzyme A/B, etc).
    - Blood samples will be obtained at the time of transplantation and the later scheduled appointments indicated above.
  - Study of immune cell activation (CD25, FOXP3, CD4, CD3, CD8, CD45RO, perforin, granzyme A/B, etc) and cytokines (IL-2, TNF-a, IFN-g, IL-10, IL-12 etc).
  - Compare biomarkers in serum, indicative of acute rejection, by at least weekly blood sampling post-Tx (RNA microarray on a series of genes related to rejection, quantitative PCR on selected genes) (28-29).
  - Study of donor specific antigens (DSA)
    - DSA present pre-Tx (already routine)
    - The evolution of de novo DSA (dnDSA), with samples taken at 6 weeks, 12 months (concurrent with scheduled biopsies) and 24 months post-Tx – as well as when indication biopsies are performed.

**2.4 Endoscopic mucosal imaging and ultrasound**

- During upper endoscopy for scheduled biopsies (see 2.3 above), pictures of the transplant duodenal mucosa will be taken.

- The mucosal images will be rated regarding rubor, edemea villous

atrophy etc. and compared to biopsy rejection scores.

- Endoscopic ultrasound (EUS) will be used when taking biopsies of the pancreas. EUS images will be sampled and stored, for comparative analysis.

- The EUS analysis will mainly involve circulatory parameters.

**2.5 Donor and recipient baseline characteristics**

- We will investigate relationships between the below mentioned donor/recipient characteristics and graft survival/surgical complications/non-surgical complications.
- Donor age
- Donor gender
- Donor BMI
- Recipient age
- Recipient gender
- Recipient BMI
- Recipient PRA (Panel Reactive Antibody) status
- Recipient comorbidity status; particularly cardiovascular status

**2.6 Non-immunological rejection markers**

- The following analyses will be performed and correlated to rejection, functional parameters (glucose levels/need for insulin (P) and creatinine (K)) and graft survival.

– By daily blood samples during the first 10 days, thereafter 3 times a week until week 10. Plasma from these samples will be frozen – to allow for subsequent analyses. .

- - Amylase (pancreas specific amylase)
  - Lipase
  - CRP
  - Amylase/Lipase/CRP combined parameter
  - C-peptide and C-peptide/Glucose/Creatinine-ratio (C-peptide : Glucose x Creatinine)
  - Pancreas Auto-Antibodies

In addition, amylase in drainage fluid will be measured daily, until the drains are removed (usually at day 4-8 post-Tx).

**2.7 Pancreas transplant monitoring with microdialysis catheters**

- At end of surgery totally three microdialysis catheters will be inserted. The catheters membranes have a 100 kDa pore size, an outer diameter of 0.6 mm and a length of 30 mm. A secure thread is positioned 60 mm from the tip (CMA 65, CMA Microdialysis AB, Stockholm, Sweden).The left abdominal wall will be punctured at three different locations close to each other by a hypodermic needle (Sterican® 14G X 3 1/8 “, B. Braun AG, Melsungen, Germany) through which the microdialysis catheter will be led. After removing the hypodermic needle one microdialysis catheter will be sutured to superfluous connective tissue on the pancreas graft, one will be sutured to the fascia of the right psoas muscle implying that the pancreas graft will be in direct contact with the catheter, and one catheter will be secured close to the duodenoduodenostomy. The catheters will be secured to the skin using a 4.0 thread. Each catheter will be perfused with 6 % hydroxyethyl starch at a rate of 1 µL/minute by microinjection pumps (CMA 107, CMA Microdialysis AB, Stockholm, Sweden), and the samples will be collected in microvials (CMA Microdialysis AB, Stockholm, Sweden)
- Postoperatively the microdialysis samples will be analyzed every second hour at the bedside for glycerol, glucose, lactate, and pyruvate using a microdialysis analyzer (Iscus Flex, CMA Microdialysis AB, Stockholm, Sweden).The lactate to pyruvate ratio will be calculated. Thereafter the samples will be frozen to -20°C and later to -80°C.
- Since this study is the first of its kind and threshold values for detecting e.g. pancreatic graft ischemia, leakages, and rejections have not been established we will not implement e.g. a decision tree in the protocol. However, since not only the physicians responsible for the studies, but also the nurses at ward have substantial experience with the microdialysis method, we expect that deviations from the normal course may lead to prescription of e.g. ultrasound Doppler examinations, and that graft complications thereby may be detected earlier. From a purely scientific point of view one could argue that blinding nurses and physicians for the results would be appropriate. However, we consider this not feasible since the researchers also are responsible for treating the patients and it is important that they have immediate access to the data to reveal e.g. inappropriate sampling.
- The catheters will be left in situ for as long as they are able to sample appropriately, or until the patient is dismissed. The patient may at any time point request removal of the catheters. In our liver studies the patients had well-functioning catheters for a median of 10 days. The catheters are easily pulled out after loosen the skin secure. According to our experience from the liver- and Whipple studies removing the catheters is not associated with any risk or pain. After study completion the samples will be thawed and analyzed en bloc for a wide range of inflammatory mediators including complement factor 5 a and the chemokine CXCL-10, which were specific biomarkers for ischemia and rejection respectively in our previous liver study. These analyses will be performed in Professor Mollnes’ lab in the Department of Immunology at our hospital. We will also consider analyzing samples for presence of various micro-RNAs.

**3 STUDY DESIGN**

This is a prospective, single cohort observational study, aimed at using a historical control group as comparison. It will be conducted at our single, national center for organ transplantation in Oslo. All pancreas recipients > 18 years of age, who fulfill the inclusion criteria, will prior to transplantation be asked for inclusion.

#### 4 DURATION OF STUDY

All consecutive PTx recipients during 2-3 years are planned to be enrolled, with an intented number of 60-80 patients. The study will continue until all patients have completed a minimum of 60 months of follow-up or have discontinued participation in the study.

**5 NUMBER OF PATIENTS**

60-80 patients will be enrolled in the study and all will receive the standard quadruple immunosuppressive regime with reduced CS dosing compared with previous cohorts (according to newly changed routine protocol).

####

#### SELECTION OF PATIENTS

- 1. **Inclusion Criteria**Patients will be eligible for study entry if **ALL** of the following criteria are met:
     1. Age ≥18 years
     2. Patients who receive a primary or secondary pancreas transplant, with or without a simultaneous kidney transplant (SPK).
     3. Women who are of childbearing potential must have a negative serum pregnancy test at baseline.
     4. Operability has to be ascertained by preoperativeexamination, performed by nephrologist, transplant surgeon and anesthesiologist.
     5. Signed and dated informed consent form.
  2. Exclusion Criteria

Patients will not be eligible if **ANY** of the following criteria are met:

- - 1. Evidence of systemic infection
    2. Presence of unstable cardiovascular disease.
    3. Malignancy < 5 years prior to entry into the trial (with the exception of adequately treated basal cell or squamous cell carcinomas of the skin).
    4. Panel-reactive antibodies (PRA) > 20% or the presence of donor-specific antigens (DSA).
    5. Any positive test for HBV, HBC or HIV.

**7 DOSAGE AND ADMINISTRATION**

**7.1 Immunosuppression**
The single cohort study group will receive our routine immunosuppressive regimen based on ATG, tacrolimus, mycophenolate mofetil and corticosteroids as follows:

7.1.1 *ATG (Thymoglobulin):*
Initiated at day 0 (the first dose preop.) at a dose of 2.5 mg/kg i.v.. Later dosing is directed by T-cell counts once daily. The T-cells are kept suppressed for 10 days post-Tx, and new doses of 1.0-2.5 mg/kg i.v. is given whenever the T-cell count rises above 0.050 x10^9^. Altogether, 2-4 doses of ATG are usually needed.

7.2.1.1 *T-cell counts*

Whole blood T-cell counts will be obtained daily from day 1-10.

7.1.2 *Tacrolimus:*

Initiated at day 0 (the first dose preop.) at a dose of 0.06 mg/kg x 2 p.o., later adjusted to achieve steady state whole-blood trough levels as follows:
Month 1-3 8-12 ng/ml
Month 3-6 4-8 ng/ml

7.1.2.1 *Tacrolimus concentration determination*
Whole blood trough concentrations for tacrolimus will be obtained daily from day 1-5, thereafter at least 3 times weekly. Concentrations will also be determined at the time of any serious adverse event.

7.1.3 *Mycophenolate mofetil (MMF):*
MMF will be given 1000 mg twice daily. It can be reduced to 750 mg twice daily in case of adverse events and further down to 500 mg in case of persisting adverse events.

7.1.4 *Corticosteroids:*
Day 0 (perop.): Methylprednisolone 250 mg i.v.

Day 1-14: Prednisolone 20 mg x 1 p.o.

Day 15-28: Prednisolone 15 mg x 1 p.o.

Day 29-60: Prednisolone 10 mg x 1 p.o.

Day 61- 180: Prednisolone 7.5 mg x 1 p.o.

Day 181 - : Prednisolone 5 mg x 1 p.o.

7.2 Concomitant Treatments

7.2.1 Required treatment

i) Prophylaxis against the development of *Pneumocystis carinii*, with trimethoprim-sulfa is required for all patients during the first 6 months

of treatment.
 ii) Prophylaxis against Cytomegalovirus (CMV) with valganciclovir for 3 months, if the donor is CMV + and the recipient is CMV ÷.

By all other CMV constellations, *preemptive* valganciclovir treatment is given, based on weekly CMV-PCR analyses (cut off: CMV-PCR count > 0).

iii) Antibiotic prophylaxis with meropenem (2 doses) and vancomycin (1 dose) at day 0.

iv) Proton pump inhibitor (pantoprazol/esomeprazole) is given for at least 2 months post-Tx.

7.2.2 Prohibited treatment

i) Investigational study drugs

ii) NSAID’s should be avoided

iii) Terfenadine, cisapride, astemizole, pimozide, cimetidine and

ketoconazole are not allowed.

**8 TREATMENT OF ACUTE REJECTION EPISODES**

P-, tD and K-biopsies must be examined in all suspected cases of rejection. This investigation should be performed before anti-rejection therapy is commenced, or at least within 24 hours of start of treatment. Whenever possible, anti-rejection therapy should be postponed until a histological diagnosis of rejection is confirmed. Acute rejection should be treated with boluses of methylprednisolone first-line according to our local practice for both pancreas- and Kidney-Tx. For *steroid resistant rejections* (defined as: no pancreas/kidney functional improvement after at least 4 boluses of methylprednisolone or rejection in repeat D-, P- or K-biopsies), ATG therapy should be initiated and administered for 7-14 days. Some cases of severe rejection may be treated with ATG primarily – also to reduce the prodiabetic steroid load.

Hyperglycemia caused by steroid dosing will be treated by subcutaneous injections of insulin, alternatively iv insulin infusion, according to current guidelines at the hospital.

All biopsies will be examined at our local pathology unit; the pancreas and duodenal biopsies by prof. Tor Jacob Eide and dr. Krzysztof Grzyb; the kidney biopsies by prof. Helge Scott and dr. Erik Heyerdahl Strøm.

**9 ADVERSE EVENTS**

All adverse events will be recorded in the appropriate section of the case record form (CRF), regardless of whether or not they are assumed to be related to the applied treatment. The nature of adverse event, details or severity, together with the date of onset, duration and outcome will be recorded. The investigator’s opinion on the relationship of the adverse event to the treatment will also be recorded.

**9.1 Definitions**
An adverse event is any adverse change from the patients baseline (pre-Tx) condition, including intercurrent disease(s) occurring during the course of the study after the treatment has started, whether considered related to treatment or not. Treatment includes all agents administered during the course of the study.

Clinical adverse events must be graded on a three-point scale (mild, moderate and severe) and be reported in the appropriate sections of the case record form.

**Mild:** Awareness of symptoms but easily tolerated

**Moderate:** Discomfort enough to interfere with normal activities

**Severe:** Completely prevents normal activities

The relationship between the adverse event and the treatment must also be assessed as follows:

**Definite:** The experience meets the following criteria:

- followed a reasonable temporal sequence from drug administration
- compatible with known drug profile
- abated upon discontinuation of the drug (dechallenge)
- with or without documentation that the experience was confirmed by reappearance of the reaction on repeat exposure (rechallenge)

**Probable:** The experience meets one or more of the following criteria:

- follows a reasonable temporal sequence from drug administration
- compatible with known drug profile and cannot be reasonably explained by the known characteristics of the patient’s clinical state
- with or without documentation that the experience abates upon

discontinuation of the drug (dechallenge)

**Possible:** The experience meets the following criteria:

- follows a reasonable temporal sequence from drug administration
- could have been produced by the patient’s clinical state or by the drug in question
- compatible with known drug profile

**Remote:**

- It is not likely to be any reasonable association between the drug and the observed experience

**Definitely Not:**

- The experience is definitely produced by the patient’s clinical state,or by other modes of therapy administered to the patient and not due to the administration of the drug

**Unknown:**

- Information provided is insufficient for a confident drug relationship to be classified

**Pre-existing Condition:**

- In this trial, a pre-existing condition (i.e., a disorder present before the adverse event reporting period started and noted on the pre-treatment medical history/physical examination form) should not be reported as an adverse event unless the condition worsens or episodes increase in frequency during the adverse event reporting period.

**9.2 Serious Adverse Event**Any clinical adverse experience or abnormal laboratory test value that is SERIOUS (including life-threatening surgical complications, grave rejection episodes, death), occurring during the course of the study, irrespective of the treatment received, have to be recorded and highlighted in our study database.

A serious adverse event is any untoward medical occurrence that, at any dose:

- Results in death

- Is life-threatening (immediate risk of death as the event occurred)

- Requires in-patient hospitalization or prolongation of existing hospitalization

- Results in persistent or significant disability/incapacity (a substantial disruption in a person´s ability to conduct normal life functions)

- Is a congenital anomaly/birth defect

- Is an overdose (whether by accident or deliberate)

- Is a significant hazard to the patient or requires intervention to prevent a serious outcome.

- Pregnancy will be recorded in the same time frame as serious AEs.

**9.3 Serious Unexpected Adverse Drug Reaction (ADR)**

A Serious Unexpected adverse drug reaction is defined as reaction which in the opinion of the Investigator is thought to be definitely, probably or possibly drug related and has not previously been known to occur for that drug either from the literature, adverse event listings or Investigator experience - but is not a common clinically insignificant illness.

**9.4 Follow Up of Adverse Events**

Any abnormal laboratory values, abnormal clinical findings and adverse events which are of clinical significance, in the opinion of the investigator, must be followed with appropriate medical management until resolved.

Individual patients will be excluded from the study if serious adverse effects related to the treatment is observed.

**10. ANALYSIS OF RESULTS**

Even though this is a single cohort observational study (SCOS), we will demonstrate power considerations involving a historic control (HC) group - as if it was a randomized study.

The primary end-points ‘Incidence of rejection’ and ‘Rate of surgical complications’ are both categorical parameters and can be treated similarly in power calculations.

**10.1** **Strategy regarding 0-hypothesis**

Regarding the ‘*Incidence of rejection*’, a non-inferior approach seems reasonable. The most important aspect is to avoid the following (Type 1) error: Not detecting an elevated rejection rate in the single cohort observational study group (SCOS), when there in fact is a difference in favor of the historic, control (HC) group, with high-CS dosing.

One could argue to use a one-sided test/“rejection region”, because it is highly unlikely that the low-immunosuppressive regimen will yield a lower incidence of rejection. However, according to statistical tradition, a two-sided test will be demonstrated.

Thus, the *0-hypothesis* will have to be:

H0: *The SCOS group will have a significantly different rejection rate compared to the HC group* (*SCOS Gr.* ≠ *HC Gr.*)

- Between groups comparison of total biopsy-proven rejection rate; based on scheduled and ‘ad hoc’ biopsies from the duodenal segment, pancreas and kidney.

-- Biopsy-proven rejection in either organ (for SPK) contributes to the rejection incidence.

Regarding the ‘*Rate of surgical complications*’, the most important aspect is to avoid the following (Type 1) error: Demonstrating an elevated complication rate in the HC group, when there in fact is no difference.

Also here, one could argue to use a one-sided test/“rejection region”, because it is no reason to believe that the low-immunosuppressive regimen will yield a higher rate of complications. However, according to custom, a two-sided test will be demonstrated. Thus, a natural *0-hypothesis* will be:

H0: *The SCOS group and the HC group will have similar rates of surgical complications* (*SCOS Gr. = HC Gr.*)

- Between groups comparison of surgical complication rate; defined as the fraction of patients experiencing one or more surgical complications, involving reoperation or reintervention.

**10.2 Sample size and Power calculation**

These statistical deductions are based on the ***binomial*** distribution/response and a two-sided test (30, 31):

Pn (1-Pn) + Ps (1-Ps) x c

N patients required per arm: (Pn – Ps)^2^

Pn: Reference probability; Ps: Probability to be detected; c: Test constant

The assumed reference rates (Pn) for the primary end-points (both actually about 30%) are based on recent data from Norway (12).

By convential presumptions; Power 1-β = 80% (🡺 c = 7,9), and 33% relative change in rejection/complication rates to be detected, these will be the figures:

| End-point | **Rejections** | **Surgical complications** |
| --- | --- | --- |
| Null hypothesis to be tested | H0: SCOS Gr.≠ HC Gr.  H1: SCOS Gr. = HC Gr. | H0: SCOS Gr. = HC Gr.  H1: SCOS Gr. ≠ HC Gr. |
| Statistical model | Binomial distribution; two-sided test | |
| Assumed reference rate (Pn) | 0.30 | 0.30 |
| Effect to be detected (Ps) | ≥ 0.40 (≥ 33% increase) | ≤ 0.20 (≤ 33% decrease) |
| Type I error (α) | 5% | |
| Power (1-β) 🡺 c | 80% 🡺 7,9 | |
| Number of pats required | 355 per arm | 292 per arm |

If we significantly release on the statistical presuppositions/demands - by only claiming 60% Power (🡺 c = 5,4) and 100% relative change in detected rejection/complication rates - these will be the figures:

| End-point | **Rejections** | **Surgical complications** |
| --- | --- | --- |
| Null hypothesis to be tested | H0: SCOS Gr.≠ HC Gr.  H1: SCOS Gr. = HC Gr. | H0: SCOS Gr. = HC Gr.  H1: SCOS Gr. ≠ HC Gr. |
| Statistical model | Binomial distribution; two-sided test | |
| Assumed reference rate (Pn) | 0.30 | 0.30 |
| Effect to be detected (Ps) | ≥ 0.60 (≥ 100% increase) | ≤ 0.15 (≤ 100% decrease) |
| Type I error (α) | 5% | |
| Power (1-β) 🡺 c | 60% 🡺 5.3 | |
| Number of pats required | 27 per arm | 80 per arm |

*Practicability with regard to statistics:*

1. It is totally unrealistic – for any Tx-center in the world – to include 600-700 PTx patients, during any reasonable time frame. In Oslo, we are by far the highest volume center in Scandinavia. Our 28 PTx’s performed in 2012 represent 5.6 p.m.p. (per million population), which actually is far higher than any other country in the world, according to figures presented by the Council of Europe in cooperation with the Spanish Tx organization (32). Even if we cooperated/coincluded with all the other PTx centers in Scandinavia (Uppsala/Göteborg/Helsinki), the potential would not exceed 50 patients annually.
2. The maximally realistic number of PTx patients to be included in Oslo during a reasonable time frame (2-3 years) will be 60-80.
3. Thus, our intentions with regard to statistical Power have to be more modest. The above figures (lower table) do however show that a doubled rejection rate in the trial arm can be detected at 60% Power with 27 x 2 = 54 patients.
4. The prospects/visions of this study consist of a lot more than detecting significant changes in rejection/complication rates. Please, cfr. paragraphs 2.2 – 2.6 of this protocol. The simultaneous biopsy strategy (D- + P- + K-biopsies) is unique. And the ‘molecular biology’ analyses of these simultaneous biopsies and blood samples have the potential to provide new insights. Furthermore, “new” potential rejection markers (C-peptide; CRP/Amylase/Lipase combined parameter) will be explored.

**10.3 Statistical methods in data analysis**

- The primary evaluation criteria ‘Incidence of rejection’ and ‘Rate of surgical complications’ will be evaluated for several populations:

- All patients who receive at least one dose of study medication, defined as the ‘Intention-to-Treat’ population.

- All patients who complete 12 months of intended study medication, defined as the ‘Intention completed’ population.

The analysis of these categorical parameters will consist of:

1. Comparisons of groups using the Fisher exact test.

2. Confidence intervals of 95% of the percentage of incidence of these

events.

- The loss of grafts and deaths will be analysed by the Kaplan-Meier method for estimating the time to events.
- Continuous (non-categorical) variables will be analyzed by student t-tests and chi-square tests.
- Microdialysis data are typically non-normally distributed. We will therefore use the Wilcoxon Signed rank test for repeated measurements and the Mann-Whitney U test for comparison of groups. We will also use linear mixed model analyses on log10 transformed data for repeated measurements. Receiver operating curves and contingency tables will be used to explore sensitivity and specificity.
- Any other methods that are not planned can be considered as alternative methods.

**10.4 Provisional analysis**

Summaries of provisional data will be carried out (descriptive statistics, graphs) during the course of the study when it is considered necessary, particularly with regard to the rejection rate. In any case, an intermediary/provisional analysis will be performed when the first 20 patients have completed 3 months follow-up. These summaries will be used only for control purposes and will not necessarily include formal statistical analyses.

Discontinuation of the study and our routine CS-dosing will be considered, at least at the 20 patients/3 months follow-up point of time, according to these criteria:

- If the biopsy-proven combined rejection rate (pancreas + duodenal-segment + kidney) in the study group is ≥ doubled compared with HC.
- If the rate of surgical complications/reoperations in the study group ≥ doubled compared with the HC.

### **11. DATA HANDLING AND RECORD KEEPING**

**11.1 Case Report Forms**

a) All data from each included patient should be recorded on case report forms (CRF’s), separate from the hospital files. Ballpoint pens will be used.

b) The investigator must also submit all incomplete case report forms that reflect patient experience with the given treatment, including retrievable data on patients who withdrew before completion of the study.

**11.2 Record Retention**

The investigator must arrange for the retention of the subject identification codes for at least 15 years after the completion or discontinuation of the trial. Subject files and other source data must be kept for the maximum period of time permitted by the hospital.

**12 ETHICAL CONSIDERATIONS**

**12.1 Institutional Review Board (IRB)/Ethics Committee (EC)**

It is the responsibility of the investigator to obtain approval of the trial protocol/ amendments from the IRB/EC before commencement of the study. All correspondence with the IRB/EC should be filed by the investigator.

**12.2 Informed Consent**

It is the responsibility of the investigator to give each subject (or the subject´s acceptable representative) prior to inclusion in the trial, full and adequate verbal and written information regarding the objective and procedures of the trial and the possible risks involved. The subjects must be informed about their right to withdraw from the trial at any time. Written subject information must be given to each subject before enrolment. Furthermore, it is the responsibility of the investigator to obtain signed informed consent from all subjects prior to inclusion in the trial.

**12.3 Declaration of Helsinki**

This study will be conducted in accordance with the Declaration of Helsinki.

**12.4 Good Clinical Research Practice (GCP)**

The study will be performed in accordance with the European ‘Guidelines on Good Clinical Research Practice’ (Consolidated guideline, CPMP/ICH/135/95).

**12.5 Unanticipated Problems**

Any changes in the study or unanticipated problems involving risks to subjects must be reported promptly to the Ethics Committee.

**13 PUBLICATIONS**

Upon completion of the study, the investigators will seek to publish the results in recognized scientific journals, within the field of transplantation.

**14 REFERENCES**

1. Kelly WD et al. Allotransplantation of the pancreas and duodenum along with the kidney in diabetic nephropathy. *Surgery* 1967;61:827-37.

2. Gruessner AC. 2011 update on pancreas transplantation: comprehensive trend analysis of 25,000 cases followed up over the course of twenty-four years at the International Pancreas Transplant Registry (IPTR). *Rev Diabet Stud* 2011;8:6-16.

3. Sutherland DE et al. Lessons learned from more than 1,000 pancreas transplants at a single institution. *Ann Surg* 2001;233:463-501.

4. Sollinger HW et al. One thousand simultaneous pancreas-kidney transplants at a single center with 22-year follow-up. Ann.Surg. 2009;250:618-30.

5. Wolfe RA et al. Comparison of mortality in all patients on dialysis, patients on dialysis awaiting transplantation, and recipients of a first cadaveric transplant. *N Engl J Med* 1999;341:1725-30.

6. Tonelli M et al. Systematic review: kidney transplantation compared with dialysis in clinically relevant outcomes. *Am J Transplant* 2011;11:2093-109.

7. Brekke IB: Indications and results of pancreatic transplantation: The Oslo experience 1983-1990. *Diabetologia* 1991;34 Suppl 1:S18-20.

8. Bentdal Ø, Fauchald P, Brekke IB, Holdaas H, Hartmann A: Rehabilitation and quality of life in diabetic patients after successful pancreas-kidney transplantation. *Diabetologia* 1991;34 (Suppl 1):S158-9.

9. Brekke IB: Duct-drained versus duct-occluded pancreatic grafts: a personal view. [Review] *Transplant International* 1993;6(2):116-20.

10. Brekke IB, Bentdal Ø, Pfeffer P, Lien B, Sødal G, Holdaas H, Fauchald P, Jervell J: Pancreastransplantasjon. Et tiårsmateriale. *Tidsskr Nor Lægeforen* 1995;115(6):703-705.

11. Brekke IB: Pancreastransplantasjon - en oversikt. *Tidsskr Nor Lægeforen* 1999;119(22):3305-3309.

12. Horneland R, Leivestad T, Jenssen T, Øyen O: Higher Donor Age And Male Recipient Gender Have A Negative Impact On Pancreas Transplant Outcomes (Surgical Complications And Graft Survival). *Transplantation*. 2012;90 (25) (Suppl 2):352.

13. Margreiter C, Aigner F, Resch T, et al. Enteroscopic biopsies in the management

of pancreas transplants: a proof of concept study for a novel monitoring tool. *Transplantation* 2012;93:207–213.

14. Mittal S, Page SL, Friend P J, Sharples EJ, Fuggle SV: De novo donor-specific HLA antibodies: biomarkers of pancreas transplant failure. *Am J Transplant.* 14(7): 1664-1671.

15. Ungerstedt U: Microdialysis - a new technique for monitoring local tissue events in the clinic. *Acta Anaesthesiol Scand*. Suppl 1997; 110-123.

16. Hillered L, Vespa PM, Hovda DA: Translational neurochemical research in acute human brain injury: the current status and potential future for cerebral microdialysis. *J Neurotrauma*. 2005; 22(1):3-41.

17. Waelgaard L, Pharo A, Tonnessen TI, Mollnes TE: Microdialysis for monitoring inflammation: efficient recovery of cytokines and anaphylotoxins provided optimal catheter pore size and fluid velocity conditions. *Scand J Immunol*. 2006; 64(3):345-52.

18. Waelgaard L, Thorgersen EB, Line PD, Foss A, Mollnes TE, Tonnessen TI: Microdialysis monitoring of liver grafts by metabolic parameters, cytokine production, and complement activation. *Transplantation*. 2008; 86(8):1096-103.

19. Haugaa H, Thorgersen EB, Pharo A, Boberg KM, Foss A, Line PD et al.: Inflammatory markers sampled by microdialysis catheters distinguish rejection from ischemia in liver grafts. *Liver Transpl*. 2012; 18(12):1421-9.

20. Haugaa H, Thorgersen EB, Pharo A, Boberg KM, Foss A, Line PD et al.: Early bedside detection of ischemia and rejection in liver transplants by microdialysis. *Liver Transpl*. 2012;18(7):839-49.

21. Haugaa H, Almaas R, Thorgersen EB, Foss A, Line PD, Sanengen T et al.: Clinical experience with microdialysis catheters in pediatric liver transplants. *Liver Transpl*. 2013;19(3):305-14.

22. [Rogers J](http://ovidsp.uk.ovid.com/sp-3.7.1b/ovidweb.cgi?&S=KDLBPDIJEIHFOFACFNPKAHEGLEMGAA00&Search+Link="Rogers+J".au.), [Farney AC](http://ovidsp.uk.ovid.com/sp-3.7.1b/ovidweb.cgi?&S=KDLBPDIJEIHFOFACFNPKAHEGLEMGAA00&Search+Link="Farney+AC".au.), [Al-Geizawi S](http://ovidsp.uk.ovid.com/sp-3.7.1b/ovidweb.cgi?&S=KDLBPDIJEIHFOFACFNPKAHEGLEMGAA00&Search+Link="Al-Geizawi+S".au.) et al.: Pancreas transplantation: lessons learned from a decade of experience at Wake Forest Baptist Medical Center. [Review]

*The Review of Diabetic Studies* 2011;8(1):17-27.

23. Meier-Kriesche HU et al. Immunosuppression: evolution in practice and trends, 1994- 2004. Am.J.Transplant. 2006;6:1111-31.

24. Schulz T, Flecken M, Kapischke M, et al. Single-shot antithymocyte globuline

and daclizumab induction in simultaneous pancreas and kidney transplant

recipient: three-year results. *Transplant Proc* 2005;37:1818–1820.

25. Boggi U, Vistoli F, Amorese G, et al. Results of pancreas transplantation

alone with special attention to native kidney function and proteinuria in type 1

diabetes patients. *Rev Diabet Stud* 2011;8:259–267.

26. Öllinger R, Margreiter C et al.: Evolution of Pancreas Transplantation

Long-Term Results and Perspectives From a High-Volume Center. *Ann Surg* 2012; 256:780-787.

27. Wu T, Abu-Elmagd K, et al. A schema for histologic grading of small intestine

allograft acute rejection. *Transplantation* 2003;75:1241–1248.

28. Li L, Khatri P et al.: A peripheral blood diagnostic test for acute rejection in renal transplantation. *Am J Transpl* 2012; 12:2710–2718.

29. Allison SJ: Transplantation; Biomarkers in peripheral blood detect acute rejection. *Nature Reviews Nephrology* 2012; 8(12):681.

30. Laake P, Olsen BR, Benestad HB (eds): Forskning i medisin og biofag. Kap.9. 2008 (2^nd^ edition) *ISBN***:** 9788205384873.

31. Binomial distribution ‘Power/Sample size’ calculator: <http://www.stat.ubc.ca/~rollin/stats/ssize/b2.html>

32. Newsletter Transplant, Vol. 17 (No 1), Sept. 2012:

http://www.transplant-observatory.org/Documents/NEWSLETTER2012.pdf

**15** STUDY FLOW CHART

|  | **BASELINE**  **DAY 0** | **Day**  **4** | **DAY**  **10** | **WEEK**  **3** | **WEEK**  **6** | **WEEK**  **10** | **MONTH**  **3** | **MONTH**  **6** | | **MONTH**  **12+24+36+60** |
| --- | --- | --- | --- | --- | --- | --- | --- | --- | --- | --- |
| Low-dose Corticosteroids | **------------------------------------------------------------------------**Continuously, tapered**-------------------------------------------------------** | | | | | | | | | |
| Mycophenolate mofetil | **-----------------------------------------------------------------------------**Continuously**------------------------------------------------------------** | | | | | | | | | |
| Tacrolimus | **-----------------------------------------------------------------------------**Continuously**------------------------------------------------------------** | | | | | | | | | |
| Thymoglobulin | **X** | | |  |  |  |  |  | |  |
| PCP-prophylaxis | **------------------------------------------------------------------------------**Continuously**--------------------------------------------** | | | | | | | |  | |
|  |  |  |  |  |  |  |  |  | |  |
| Medical History | **X** |  |  |  |  |  |  |  | |  |
| Previous Treatment | **X** |  |  |  |  |  |  |  | |  |
| Physical examination incl. vital signs | **X** |  |  | **X** |  |  | **X** | **X** | | **X** |
| PA Chest X-ray | **X** | To be obtained when clinically indicated | | | | | | | | |
| Complete blood count | **X** | **X** | **X** | **X** | **X** | **X** | **X** | **X** | | **X** |
| Fasting Blood Chemistries incl. amylase, lipase, CRP | **X** | **3 days a week** | | | **X** | **X** | **X** | **X** | | **X** |
| Blood T-cell counts | **Daily** | | |  |  |  |  |  | |  |
| Blood C-peptide, HbA1c, Auto-Ab | **3 days a week** | | | | **X** | **X** | **X** | **X** | | **X** |
| Microdialysis monitoring w/ catheters | **X** | | |  |  |  |  |  | |  |
| Blood fasting total, LDL and HDL-cholesterol and triglycerides | **X** | **X** | **X** | **X** | **X** | **X** | **X** | **X** | | **X** |
| Scheduled endoscopic (EUS) biopsies | **tD + nD** |  |  | **P + tD + nD** | **P + tD+nD** |  |  |  | | **P + tD+nD** |
| Conventional percutaneous US-guided biopsies | **K** |  |  |  | **P + K** |  |  |  | | **P + K (12)** |
| Blood immunology: RNA arrays.  Quantitative RT-PCR | **X** | **X** | **X** | **X** | **X** | **X** | **X** | **X** | | **X(12+24)** |
| HLA Donor specific antigens (DSA) | **X** |  |  |  | **X** |  |  |  | | **X (12+24)** |
| CMV-PCR | **X** |  |  | **X** |  |  | **X** | **X** | | **X** |
| Pregnancy Test | **X** | Whenever clinically indicated | | | | | | | | |
| Concomitant Medication | **X** | **X** | **X** | **X** | **X** | **X** | **X** | **X** | | **X** |
| Tacrolimus Trough Levels |  | **Week 1** | **Week2+ 3** | **X** | **X** | **X** | **X** | **X** | | **X** |
| MMF Through Levels | **X** | **X** | **X** | **X** | **X** | **X** | **X** | **X** | | X |
| Adverse Event Monitoring |  | **X** | **X** | **X** | **X** | **X** | **X** | **X** | | **X** |
